# Sclerosing Mesenteritis Presenting as Intestinal Occlusion: A Case Report

**DOI:** 10.7759/cureus.75235

**Published:** 2024-12-06

**Authors:** Miguel E Rodrigues, João Luís Pinheiro, Bruno Barbosa, Carolina Canhoto

**Affiliations:** 1 General Surgery, Unidade Local de Saúde (ULS) de Viseu Dão-Lafões, Viseu, PRT; 2 Esophagogastric Surgery, Unidade Local de Saúde (ULS) de Viseu Dão-Lafões, Viseu, PRT

**Keywords:** abdominal pain, histologic diagnosis, imaging, incidental finding, intestinal occlusion, sclerosing mesenteritis, surgery

## Abstract

Sclerosing mesenteritis is a chronic disease that primarily affects the mesenteric adipose tissue and encompasses a range of fibrotic and inflammatory pathologies. Due to its low incidence, the etiology remains unclear, though various factors are thought to contribute to its onset. Clinical manifestations are nonspecific, ranging from asymptomatic cases to persistent abdominal pain, which is the most common symptom.

Incidental findings on imaging have increased with the widespread use of computed tomography (CT) scans. However, the diagnosis remains histological. Sclerosing mesenteritis is mainly associated with a good prognosis, as it typically follows a benign clinical course and rarely presents with complications or persistent symptoms.

In this article, the authors present a clinical case of an 82-year-old male patient who presented with abdominal pain, constipation, and vomiting. The patient underwent an urgent laparotomy for intestinal occlusion. Histological examination confirmed sclerosing mesenteritis.

Due to its limited understanding, sclerosing mesenteritis is often misdiagnosed. It should be considered as a differential diagnosis, particularly in patients with poorly defined abdominal pain, normal laboratory studies, and nonspecific imaging findings, to avoid unnecessary treatments. Nonetheless, urgent surgical intervention may be necessary in cases presenting with intestinal occlusion or uncontrolled pain.

## Introduction

Sclerosing mesenteritis is a rare, idiopathic condition with an incidence of around 1% [1], although the true prevalence may be higher due to the increasing use of computed tomography (CT) scans worldwide [2]. It is characterized by chronic, idiopathic fat necrosis, fibrosis, and inflammation, mainly affecting the mesentery. The condition includes various subtypes, such as mesenteric lipodystrophy, retractile mesenteritis, and mesenteric panniculitis, which are distinguished only through histologic analysis [3]. Due to its rarity, the etiology and risk factors remain poorly understood, though trauma, autoimmune diseases, and malignancies have been suggested as potential contributors [4].

Treatment is rarely required. In cases of refractory pain, medical therapy with anti-inflammatory drugs, immunosuppressives, or steroids may be beneficial [5]. As mentioned, the disease can present as intestinal occlusion or ischemia, sometimes warranting urgent surgical treatment.

## Case presentation

An 82-year-old male patient presented to the emergency department with abdominal pain, bloating, vomiting, and a five-day history of constipation. Relevant medical history includes a partial gastrectomy for lymphoma 20 years ago, type 2 diabetes, and asthma. He was on appropriate medications but took no other medications.

Physical examination revealed a normotensive, hemodynamically stable patient, with no tachycardia or fever. Abdominal examination showed a soft, depressible abdomen with tenderness, primarily in the epigastric region, and no signs of peritoneal irritation. Metallic bowel sounds were noted upon auscultation.

Laboratory tests, including hemogram, coagulation profile, and blood biochemistry, were unremarkable. Immunochemical tests showed a C-reactive protein (CRP) level of 3.4 mg/dL. Arterial blood gas analysis revealed no acid-base disturbances. Lactate levels were elevated at 4 mmol/L.

**Figure 1:**
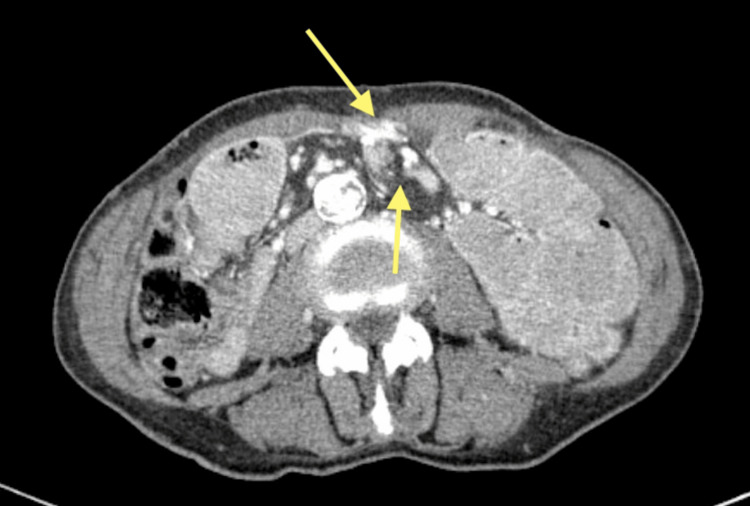
CT scan showing the transition point with intestinal rotation and a mesenteric vessel swirl. Proximal intestinal distention is seen due to rotation, accompanied by a mesenteric vessel swirl (yellow arrows). Beyond the transition point, intestinal loops are either collapsed or of normal caliber.

The CT scan revealed jejunal distension with a clear transition point with intestinal rotation and a mesenteric vessel swirl (Figure 1).

Collapsed ileum loops distal to the occlusion point were identified, and urgent laparotomy was recommended.

In the operating room, signs of a previous subtotal gastrectomy with Billroth II reconstruction were noted. A mesenteric fibrotic lesion was found at the transition point, with normal-caliber intestinal loops distal to it. Segmental enterectomy was performed with a latero-lateral manual anastomosis. No other lesions were found, and there were no postoperative complications. The patient was discharged on the third day post-surgery, clinically stable, with normal bowel movements and tolerating oral feeding.

**Figure 2:**
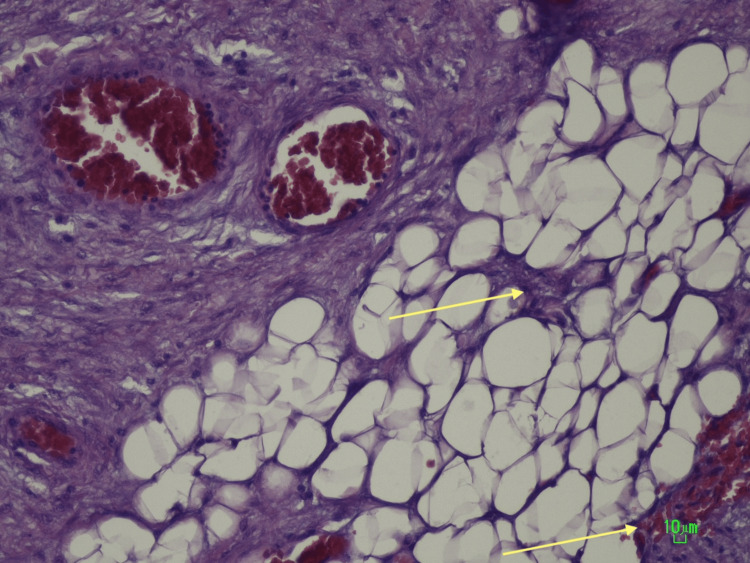
Enterectomy specimen under the microscope. Enteric segment with inflammation and focal necrotic fat (yellow arrows).

**Figure 3:**
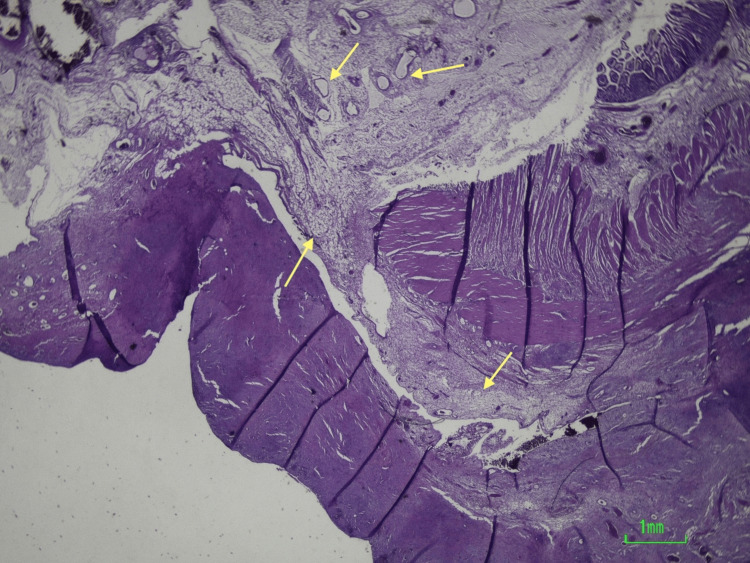
Enterectomy specimen under the microscope. Enteric segment with fat necrosis and reactive fibroblastic proliferation (yellow arrows).

Histological analysis revealed fibrotic proliferation, fat necrosis, and areas of lipodystrophy, consistent with sclerosing mesenteritis (Figure 2 and Figure 3).

The patient was followed for one year postoperatively, remaining clinically well with normal bowel movements and no weight loss. Clinical workup and blood tests showed no abnormalities. At three years post-surgery, the patient reported no further symptoms.

## Discussion

​​​​​Our case supports the notion that sclerosing mesenteritis may be linked to an abnormal healing process and connective tissue repair following tissue trauma [6].

Current studies suggest that the disease primarily affects males, with a mean age of 65, and typically involves the small bowel mesentery as the main site [3,7]. This is consistent with our case, despite the patient's slightly older age.

Current literature suggests that sclerosing mesenteritis typically presents with normal blood work, with a mildly elevated C-reactive protein [8]. In line with these studies, we observed a slight increase in CRP levels, with no other relevant abnormalities.

There are no pathognomonic findings for sclerosing mesenteritis, neither on the physical evaluation nor in imaging studies. However, two signs (described in several studies) may suggest the diagnosis, though they are observed in only a small percentage of cases. These include the “fat ring” sign, which reflects inflammation of the central mesentery while preserving a circumferential rim of fat around the mesenteric vessels, and the “tumor pseudocapsule.” No imaging signs are required for diagnosis [7-10], and neither of these signs was observed in our case.

Our findings align with existing literature in terms of epidemiology, blood work, and clinical presentation. The postoperative follow-up was also consistent with reported cases, showing complete symptomatic resolution. However, the diagnosis remains challenging, as these descriptions are not specific.

Although surgery is rarely needed, sclerosing mesenteritis presenting with intestinal obstruction requiring emergency surgery has been reported only infrequently [11]. This makes the diagnosis in the emergency setting challenging, especially given that specific imaging findings are rare and were not observed in our case. In this case, urgent treatment becomes the priority. However, the diagnosis of sclerosing mesenteritis was confirmed through histologic analyses.

While sclerosing mesenteritis is often a diagnosis of exclusion, revisiting the clinical history (including past surgeries and trauma), illness course, and imaging findings may help avoid unnecessary invasive procedures in select patients [12].

As mentioned, intestinal occlusion or noncontrolled abdominal pain may warrant an urgent surgical intervention. Mesenteric trauma, which may contribute to the disease’s etiology, is also associated with significant complications.

## Conclusions

The authors suggest that in patients with nonspecific, poorly characterized, and recurrent abdominal pain or sudden occlusive symptoms with no specific abnormalities in blood analysis, sclerosing mesenteritis should be considered as a differential diagnosis. While directed treatment is typically not required, suspecting this condition may help identify patients who could benefit from a trial of medical therapy. However, it is important to note that intestinal occlusion requires urgent treatment, which takes precedence and delays the diagnosis until histological analysis.

The literature primarily consists of single-case reports, as randomized, multicenter trials are lacking due to the disease’s rarity. Continued reporting of these cases is essential to better understand this relatively unknown condition, ultimately improving outcomes and reducing morbidity.
